# Exploring patient-centered aspects of home care communication: a cross-sectional study

**DOI:** 10.1186/s12912-020-00483-1

**Published:** 2020-09-29

**Authors:** Jessica Höglander, Jakob Håkansson Eklund, Peter Spreeuwenberg, Hilde Eide, Annelie J. Sundler, Debra Roter, Inger K. Holmström

**Affiliations:** 1 School of Health, Care and Social Welfare, Mälardalen University, SE-721 23 Västerås, Sweden; 2 NIVEL (Netherlands Institute for Health Services Research), Utrecht, the Netherlands; 3 Science Centre Health and Technology, Faculty of Health and Social Sciences, University of South-Eastern Norway, Drammen, Norway; 4 Faculty of Caring Science, Work Life and Social Welfare, University of Borås, Borås, Sweden; 5 Department of Health, Behavior and Society, Johns Hopkins Bloomberg School of Public Health, Baltimore, MD USA; 6 Department of Public Health and Caring Sciences, Uppsala University, Uppsala, Sweden

**Keywords:** Communication, Home care, Patient-centered care, Registered nurses, RIAS

## Abstract

**Background:**

Communication is a cornerstone in nursing and aims at both information exchange and relationship building. To date, little is known about the naturally occurring communication between older persons and nurses in home care. Communication might heal through different pathways and a patient- or person-centered communication could be important for health and well-being of older persons. However, the delivery of individualized home care is challenged by routines and organizational demands such as time constraints. Therefore, the aim of this study was to explore the patient-centered aspects of home care communication between older persons and registered nurses.

**Methods:**

In total 37 older persons (aged 65 years or older) and eleven RNs participated in 50 audio-recorded home care visits. Roter Interaction Analysis System (RIAS) was used to code verbal communication. A ratio from these codes, establishing the degree of patient-centeredness, was analyzed using a Generalized Linear Mixed Model.

**Results:**

The present home care communication contained more socio-emotional than task-oriented communication and the emotional tone was largely positive. The global affect ratings reflected an overall positive tone (*m* = 39.88, *sd* = 7.65), with higher ratings on dimensions of, for example, responsiveness/engagement and interactivity or interest were more frequent than those that may be considered as less-positive emotions (*m* = 15.56, *sd* = 3.91), e.g. hurried, dominance or anger. The ratio of the degree of patient-centered communication in the home care visits was an average of 1.53, revealing that the communication could be considered as patient-centered. The length of the visits was the only characteristic significantly associated with the degree of patient-centeredness in the communication, with a peak in patient-centeredness in visits 8–9 min long. Sex, age or procedural focus showed no significant effects on the degree of patient-centeredness.

**Conclusion:**

Overall, the degree of patient-centeredness and a positive emotional tone, which might have a positive outcome on older persons’ health, was high. Longer visits provided a higher degree of patient-centeredness, but no linear increase in patient-centeredness due to length of visit could be observed. The findings can be used for education and training of nurses, and for providing individualized care, e.g. patient- or person-centered care.

## Background

The ageing population is increasing internationally at a rapid pace. In order to maintain the health of the ageing population, health systems need to strengthen older people’s abilities and be organized around older people’s own needs and preferences [1]. Providing care that meets the needs of older people can result in reduced loneliness and increased life satisfaction [2]. To be encountered as a unique person is important for individuals of all ages [3], especially for older persons who often experience social isolation [4, 5]. Nevertheless, the delivery of an individualized, such as patient- or person-centered, home care is challenged by routines and organizational demands such as time constraints, lack of continuity of staff and having to work according to the procedures and decisions required by the organization, which could mean doing both too much and too little for the older person [6–8]. As a result, home care has been criticized for insufficient inclusion of older persons’ individual needs, preferences, wish for personal integrity and social interactions [9, 10].

### Patient- and person-centered care

A patient- or person-centered approach is central for including the perspective of the person in need of care [11–13]. Patient-centered care and person-centered care are two concepts that have many similarities and they are often used interchangeably [12]. A literature review showed that these similarities can be found in shared attributes, such as empathy, including *compassion*, *emotional support*, and *understanding,* respect, which meant to *respect beliefs*, *respect values,* and *support dignity,* shared decision-making, communication, which meant a two-way interaction between the carer and the patient where information was being conveyed and shared, in a respectful and open manner, individuality and holistic focus [14], referring to the importance of seeing the unique individual, his/her needs, wishes, beliefs and preferences [12–14]. This requires a communication that includes active listening to the person’s narrative, as well as a partnership in which experience and knowledge are shared [12, 13]. Despite these similarities, differences have been found in the goals for patient- and person-centered care. There seems to be more of a functional dimension to patient-centered care than to person-centered care. Person-centered care aims to facilitate quality of life, including achieving life goals and values, whereas patient-centeredness often focuses on enhancing function. However, both patient- and person-centered care are found to have positive impact on the experience of quality of care and health for patients and there should be no opposition between them [14].

A patient- or person-centered communication is important but complex, and based on the core attributes of acceptance, genuineness and empathic understanding. A previous study in Swedish home care setting concluded that attributes of person-centered communication included recognizing, inviting and involving older persons. To facilitate this form of communication, attentiveness and responsiveness on the part of RNs were pivotal [15]. Whether the communication or relationship can be referred to as person-centered or not may be dependent on whether the core attributes are present in the communication [16].

### Home care for older persons

Older persons who receive home care live at home with daily help and care from their nursing staff. Home care supports older persons’ efforts to live meaningful and independent lives and forestalls the move into a nursing home or residential care [17, 18]. Many older persons wish to remain in their homes for as long as possible [19]. Receiving home care is therefore one way for older persons to continue to live at home, despite declining health [17].

Being old and receiving care in one’s own home sometimes creates a vulnerable situation as the home becomes the nursing staff’s workplace, with reduced opportunities for the older person to make decisions regarding his/her situation [20]. With increasing age, the ability to communicate changes and impacts on how older persons communicate [21]. Older persons express both existential and emotional concerns [22–24] that need to be responded to by the nursing staff, preferably in an individualized way [22].

### Communication in home care

Patients sometimes prioritize receiving information about treatment or illness over emotional or social talk, sometimes it is the opposite [25]. Hence, both the task-oriented and socio-emotional/affective communication are important features in the communication [25, 26]. Home care communication is partly influenced by the variety of tasks that needs to be performed, which influence the topic of the communication [23], but is also socio-emotional [23, 24].

The communication competence of registered nurses (RN) is crucial for establishing relationships and exchanging information [27]. Nurses are however sometimes perceived as more focused on their chores than on talking with their patients [3] and home care is often criticized for being too standardized [8–10] with different tasks conducted on a tight schedule with little wiggleroom for individual needs and wishes.

Home care can however fill emotional and social needs of older persons [4, 23, 28, 29], who might lack social contacts or seldom leave the house. The caregiver’s ability to listen, often emphasized as an important skill [16, 30, 31], can help alleviate suffering [31, 32] and have a significant impact on the experience of health [33, 34]. According to Street et al. [34], communication might heal through different pathways. Talk itself can be therapeutic, but often communication affects health outcomes more indirectly. Positive outcomes of communication include patient understanding, trust, and clinician– patient agreement. These affect intermediate outcomes such as increased adherence and better self-care skills which, in turn, affect health and well-being [34]. Personal characteristics, such as gender and age, of both patient and health care professionals, sometimes influence the communication. Previous research has shown that females are more involved in emotional communication [35, 36], and hospital visits with female healthcare providers contain more patient-centered communication [37–39]. However, there are few studies that have explored communication in home care settings, despite its importance for the increasing older population. Therefore, this explorative study of naturally occurring communication in home care was performed, to lay a ground for further large-scaled studies, interventions and education of staff.

The purpose of this study is to explore the patient-centered aspects of home care communication between older persons and registered nurses. We were especially interested in whether an explicit home care visit function, such as the performance of a medical procedure, would affect the patient-centeredness of the visit communication. With this question in mind, the following research questions were addressed: (a) What is the relative focus of home care visit communication in terms of task-focused or socio-emotional exchange? (b) What is the overall emotional tone of exchange between the RNs and the older persons in these visits? (c) What is the overall degree of patient-centeredness? and (d) Are there older person, nurse or visit characteristics that impact the degree of patient-centeredness in home care communication?

## Methods

### Design

This was an exploratory, cross-sectional study of communication in Swedish home care with older persons, and a part of the international research program COMHOME [40].

### Setting

A convenience sample of four home care institutions in mid-Sweden participated in the data collection.

### Participants

Participants in this study consisted of a convenience sample of 11 RNs and 37 older persons (65 years or older). Even though we collected data for over a year, we were unable to include more participants due to RNs working conditions and heavy workload. Inclusion criteria for the RNs were that they were Swedish speaking and employed within home care. The RNs were asked to recruit older persons for the study according to inclusion criteria. The inclusion criteria for the older persons were that they should be Swedish speaking, without speech or cognitive impairments, and receiving home care.

### Data collection

The data consisted of 50 audio-recorded home care visits, recorded from October 2014 until November 2015. These recordings include a variety of home care visits, and RNs and older persons (Tables 1 and 2) of different genders and ages. The lengths and purposes for the home care visits varied and since naturally occurring communication was collected some visits contained more dialogue or longer pauses and silences than others. Visits were distinguished by purpose, either the performance of a medical procedure, such as blood-pressure controls, blood-samples, vaccinations, wound-care or information about test-results or treatments, or the provision of routine care (e.g. medication-refills, daily status check or socialization).

**Table 1:** Sample description

	Median [sample range], or %
**RN (*n*** **= 11)**	
Sex	
Female (*n* = 8)	73
Male (*n* = 3)	27
Age	47 [39–62]
**Older persons (*n*** **= 37)**	
Sex	
Female (*n* = 29)	78
Male (*n* = 8)	22
Age	89 [65–95]
**Home care visits (*n*** **= 50)**	
Type of visit	
No-medical procedure (*n* = 34)	68
Medical procedure (*n* = 16)	32
Visit length in minutes	5 [1–31]

**Table 2:** Division of home care visits by female and male participants

	Female RN	Male RN
**Older female**	25	11
**Older male**	9	5

The RNs were instructed to start the audio recording upon entering the older person’s home and end it when leaving. Hence, the length of the visit was assessed from the time the audio recording started until it was turned off. Most of the time, the RNs greeted the older person as soon as the audio recording started and said goodbye before it was turned off. The RNs were also instructed to inform the older person that they were recording the visit. The audio recording device was worn on the RN’s upper arm. Each RN was asked to record five to ten audio recordings of their home care visits to older persons. This resulted in each RN making between two and eight recordings (*m* = 4.6, *sd* = 1.9). The older persons participated in one to four recordings (*m* = 1.4, *sd* = 0.7) per person, depending on the participating RNs’ work schedule and the older persons’ care needs. This distribution of audio recordings sometimes led to repeated observations with the same participant, which in turn resulted in clustered structures in the collected data, see section 2.6 Analysis. The time the RNs had for data collection varied depending on their work schedules and the audio recordings were collected on an ongoing basis by the researchers. Reminders were sent out after 2–3 weeks if the audio recordings dragged on.

### Measures

#### The Roter interaction analysis system

The Roter Interaction Analysis System (RIAS) [41, 42] was used for coding the audio-recorded communication in home care visits. RIAS is a widely used instrument for coding health care dialogues, applied in a variety of care contexts, and categorize elements in the communication as either socio-emotional or task-focused [42]. RIAS assign codes directly from the recording, without the need of transcription [42]. RIAS codes are applied to the smallest unit of expression to which a meaningful code can be assigned, generally a complete thought expressed as a simple sentence, a sentence clause or a single word. Codes are individually applied to the speech of the speaker; most of the system codes are applied in a parallel manner to all speakers, with a handful of codes specified as applicable to a single speaker. Codes are mutually exclusive and exhaustive, such that a statement is assigned only one code and all statements are coded [43].

A ratio of the degree of patient-centered communication was calculated following conventions established in previous studies [44]. In this calculation, the numerator consists of provider and patient categories of psychosocial and lifestyle exchange, emotional exchange and partnership/activation statements. Patients’ medical questions are also included. The denominator includes provider biomedical information and question categories, instructions and directions, and biomedical information from the patient [44].

In addition to the verbally defined codes, the RIAS assess the overall affective tone of the visit as conveyed by each speaker [43] on a 6-point Likert scale, indicating none or low signs of the affect to a good deal or high signs of the affect. This rating is made at the end of the coded visit. It is assigned separately for each speaker and designed to reflect the overall emotional impression of the dialogue. Some examples of rated affects were: anger/irritation, anxiety/nervousness, dominance/assertiveness, interest/attentiveness, friendliness/warmth, sympathetic/empathetic or hurried/rushed [43].

RIAS has demonstrated reliability and validity during a number of previous studies [42], with a general average between 0.8 and 1.0 reliability, assessed through the Pearson correlation analysis [43]. In the present study, a random sample of 10 audio recordings (20%) was selected for double coding to assess the interrater reliability of the RIAS coding. Initially, three recordings were co-coded and discussed between four of the authors (JH, JHE, AJS & IKH) to establish internal agreement. Thereafter, two of the authors (JH & IKH) separately double coded seven audio recordings (14%), which were compared, and the interrater reliability was calculated to 0.8 using the Pearson correlation.

### Analysis

A Generalized linear mixed model (GLMM), using IBM SPSS statistics 24 [45], was conducted to explore the influence of different variables on the degree of patient-centeredness in the home care communication. Analyses were carried out by the first author JH, in collaboration with author PS, who is a statistician by training, and the second author JHE, who has longstanding experience in quantitative analyses. The data from the home care visits were count data, which in this study were not normally distributed. The data were also hierarchical, and both the RNs and older persons have been recorded several times in the home care visits, resulting in clustered data. The Poisson GLMM analysis adequately accounts for the not normally distributed and clustered outcome variable (the degree of patient-centered communication). The length of the visits and the age variables were dichotomized to avoid false results caused by outliers in the data.

The GLMM started with an empty model (Model 0). Thereafter, in Model 1 and Model 2, variables related to the home care visits in terms of type of home care visit and length of the home care visit were added. Finally, characteristics of the older persons and the RNs, in terms of age and sex, were added in Model 3. In the GLMM models, the fixed effects were reported with unstandardized coefficients, together with the standard errors. The independent variables have been checked for multi-collinearity, which showed low to moderate correlations (*r* < .7). There were no reliable interactions in the models, and we have omitted interactions in the presentation of the results.

### Ethical considerations

Ethical approval was obtained from the Regional Ethical Review Board in Uppsala, Sweden (Dnr 2014/018). Oral and written information about the study was given to the participants, both RNs and older persons. The participants were further informed about how the collected data would be handled, stored and presented/published and about their rights as participants. All participants were guaranteed confidentiality and had to be able to give their written, informed consent in order to participate. Older persons with cognitive impairments were not included. All written consents were collected in sealed envelopes by the researchers before handing out the audio recording devices used for data collection. The RNs were instructed to stop the audio recording if they or the older person felt uncomfortable or requested it to be turned off during the home care visits.

## Results

### The focus of home care visits in terms of task-focused or socio-emotional exchange

In the home care visits with RNs, a total 10,028 utterances were RIAS coded, of which the RNs made 54% of the utterances and the older persons 46%. The audio-recorded home care visits between the RNs and older persons revealed that the content of the communication was multifaceted, combining social talk (*m* = 130.26, *sd* = 120.21) with task-focused or bio-medical topics (*m* = 70.30, *sd* = 56.62) (Table 3).

**Table 3:** Presence of RIAS-coded utterances in the two participant groups, divided into visits with or without the intent of a medical procedure

	Older persons N [Mean] (SD)		Registered nurses N [Mean] (SD)		Examples of RIAS utterances
	Medical procedure	Non-medical procedure	Medical procedure	Non-medical procedure	
**Socio-emotional (65%)**					
Personal or social talk (14%)	232 [14.50] (17.63)	538 [15.82] (26.70)	205 [12.81] (12.48)	469 [13.79] (14.86)	*The wedding picture was in today’s newspaper, did you see it?*
Emotional talk (concern; empathy; legitimize; reassure; ask for reassurance; Self-disclosure^a^; partnership^a^) (6%)	61 [3.81] (4.32)	128 [3.76] (5.18)	145 [9.06] (9.11)	233 [6.85] (7.74)	*I understand, if something feels wrong it affects all of you.*
Positive talk (laugh; approve; compliment; agree) (20%)	371 [23.19] (17.58)	752 [22.12] (25.61)	287 [17.94] (13.96)	601 [17.68] (18.76)	*I really enjoy listening to you!*
Negative talk (Disagree; Criticism) (2%)	45 [2.81] (3.21)	97 [2.85] (4.55)	19 [1.19] (1.83)	36 [1.06] (1.91)	*I don’t like her!*
Back-channel (23%)	160 [10.00] (7.48)	432 [12.71] (10.44)	467 [29.19] (26.14)	1235 [36.32] (42.47)	*Mmm, ok*
**Task-focused (*35%*)**					
Procedural (transition words; orientation/instruction) (4%)	60 [3.75] (3.82)	84 [2.47] (3.33)	148 [9.25] (6.27)	157 [4.62] (3.81)	*Now we will check your blood pressure*
Checks (checks for understanding; ask for understanding; bid for repetition; ask permission^a^; ask opinion^a^) (3%)	51 [3.19] (2.61)	52 [1.53] (1.73)	42 [2.63] (3.95)	141 [4.15] (5.37)	*Is that so?*
Medical questions (all questions: medical, therapeutic, other) (4%)	32 [2.00] (1.63)	84 [2.47] (2.60)	91 [5.69] (3.40)	187 [5.50] (7.05)	*Do you take stronger painkillers now?*
Psychosocial questions (all questions: lifestyle, psychosocial) (1%)	2 [0.13] (0.34)	5 [0.15] (0.44)	31 [1.94] (2.18)	77 [2.26] (3.51)	*Have you been walking outside lately?*
Medical information (all medical, therapeutic, other information; consultation medical/therapeutic^a^) (17%)	261 [16.31] (12.00)	675 [19.85] (22.44)	265 [16.56] (9.66)	473 [13.91] (11.76)	*Those pills risk making you constipated.*
Psychosocial information (all psychosocial, lifestyle information; consultation psychosocial/lifestyle^a^) (6%)	127 [7.94] (7.60)	371 [10.91] (14.25)	18 [1.13] (1.36)	76 [2.24] (2.36)	*I have to eat, if I do not want to lose more weight.*
Request for service or medication^b^ (−)	0 [0.00] (0.00)	5 [0.15] (0.44)			*Can you pass this on to the doctor when you see her?*

^a^Provider category only

^b^Patient category only

The visits were categorized according to whether the visit was intended for a medical procedure or a provision of routine care. We hypothesized that there might be differences in the frequency of the RIAS utterances (Table 3), depending on the purpose of the visit. However, only very small differences were shown between the average numbers of RIAS codes in the medical procedure visits compared to the non-medical procedure visits.

Almost twice as many visits were characterized as routine (*n* = 34), while the minority of visits provided medical procedures (*n* = 16). The average length of the home care visits differed by these functions; routine visits (e.g. medication-refills or daily status) were slightly shorter (*m* = 6.79 min, *sd* = 6.43), than visits in which medical procedures (e.g. wound-care, vaccinations or blood-pressure) were performed (*m* = 7.19 min, *sd* = 5.14).

### The emotional tone in the home care communication

The global affect ratings reflected an overall positive tone (*m* = 39.88, *sd* = 7.65), with higher ratings on dimensions of, for example, responsiveness/engagement and interactivity or interest were more frequent than those that may be considered as less-positive emotions (*m* = 15.56, *sd* = 3.91), e.g. hurried, dominance or anger (Table 4). Dividing the home care visits into visits with a medical procedure and routine care visits revealed higher ratings of positive affects (Table 4). Visits intended for a medical procedure revealed a higher positive (*m* = 42.81, *sd* 7.95) than negative (*m* = 15.56, *SD* = 3.12) affect tone. Similarly, in routine care visits, the positive affect tone (*m* = 38.50, *sd* = 7.22) was higher than the negative affect tone (*m* = 15.56, sd = 4.27).

**Table 4:** Global affect rating of the home care visits

Global affect ratings (scale of 1–6)	Non-medical visits (***n*** = 34)		Medical visits (***n*** = 16)	
	Older persons Mean (*sd*)	Nursing staff Mean (*sd*)	Older persons Mean (*sd*)	Nursing staff Mean (*sd*)
Anger/Irritation	1.97 (1.24)	1.15 (0.44)	1.06 (0.25)	1.13 (0.34)
Anxiety/Nervousness	1.21 (0.54)	1.00 (0.00)	1.31 (0.87)	1.00 (0.00)
Depression/Sadness	1.12 (0.54)	1.00 (0.00)	1.38 (1.09)	1.00 (0.00)
Emotional Distress/Upset	2.06 (1.48)	1.00 (0.00)	2.56 (1.46)	1.00 (0.00)
Dominance/Assertiveness	1.35 (0.88)	1.50 (0.99)	1.13 (0.34)	1.81 (1.22)
Interest/Attentiveness	3.47 (0.66)	3.97 (0.72)	3.69 (0.79)	4.31 (0.70)
Friendliness/Warmth	3.35 (1.30)	3.32 (1.22)	3.81 (1.33)	3.88 (1.31)
Responsiveness/Engagement	3.76 (0.78)	4.00 (0.70)	3.94 (0.85)	4.19 (0.54)
Sympathetic/Empathetic	1.15 (0.44)	1.79 (1.04)	1.06 (0.25)	2.69 (1.70)
Respectfulness	3.06 (0.60)	3.44 (0.75)	3.00 (0.63)	3.88 (0.96)
Hurried/Rushed	1.00 (0.00)	1.21 (0.64)	1.19 (0.75)	1.00 (0.00)
Interactivity	3.59 (0.89)	3.59 (0.82)	4.06 (1.00)	4.31 (0.87)

### The degree of patient-centered communication and its influencers in home care visits

The ratio of the degree of patient-centered communication in the home care visits was an average of 1.53, revealing that the communication could be considered as patient-centered. A ratio of 1 would have indicated a balance between patient-centered communication and a more bio-medical, or task-focused, communication.

When exploring possible effects on the degree of patient-centeredness in the home care communication, only length of the visit (− 0.368) showed a significant association with the degree of patient-centeredness (see Models 2 and 3 in Table 5). The results of the GLMM showed that when the home care visits were longer than 5 minutes, the degree of patient-centeredness in the communication was higher than for shorter visits. However, the degree of patient-centeredness showed no linear increase during longer visits. Adding the age and gender of the older persons and the RNs (Model 3) did not improve the fit of the model.

**Table 5:** Summary of the generalized linear mixed models of person-centered communication with binary coded explanatory variables

Variables	Model 0	Model 1	Model 2	Model 3
Fixed effects				
Intercept estimate	0.325 (0.168)	0.221 (0.182)	0.392 (0.176)	1.042 (0.540)
Type of visit (Non-medical)		0.157 (0.116)	0.205 (0.114)	0.167 (0.118)
Length of visit (≤ 5 min)			−0.389 (0.132)*	− 0.368 (0.149)*
Age older person (≤ 89 years)				−0.152 (0.168)
Age registered nurse (≤ 47 years)				−0.490 (0.367)
Gender older person (Female)				−0.080 (0.188)
Gender registered nurse (Female)				−0.364 (0.415)
Random effects				
Nursing staff variance	0.233 (0.133)	0.225 (0.130)	0.172 (0.100)	0.175 (0.121)
Older person variance	0.133 (0.053)*	0.115 (0.049)*	0.093 (0.041)*	0.112 (0.050)*
Residual	1.00	1.00	1.00	1.00
Proportion explained variance *R*^*2*^				
*R*^*2*^ nursing staff		3.43	26.18	24.89
*R*^*2*^ older person		13.53	30.08	15.79

* *p* < 0.05

Model 2 was beneficial for explaining the RN variances (*R*^*2*^ = 26.18%) and for explaining the variance in older persons (*R*^*2*^ = 30.08%). Based on these results, 73.82% of the RN variance and 69.92% of the older person variance remain unexplained by the variables under study.

## Discussion and conclusion

### Discussion

Home care communication is a limited area of research and this is, to the best of our knowledge, one of the first studies using RIAS to explore patient-centered communication in home care visits. An interesting finding was that the initial ideas about differences in degree of patient-centeredness related to focus of the visits was not supported. This indicates that whether the visits included a medical procedure or not, did not show any significant association with the degree of patient-centeredness. This is an important finding, since home care is sometimes criticized for being too task-oriented and routine based [8–10], as descibed in the introduction.

Length of visit was significantly associated with the degree of patient-centeredness, as longer visits had a higher degree of patient-centered communication. This may indicate that when the RN has more time, the communication may also become more patient-centered. After 8–9 min, the degree of patient-centeredness did not show any linear increase as the visits became longer. This peak at 8–9 min is intriguing and may be investigated in future research to explore possible reasons for the high degree of patient-centered communication. Having enough time to communicate is important for establishing a more individualized communication, but how well the time is used for communication is also important. Therefore, longer visits should not be used as a quick fix to increase patient- or person-centeredness, but as an important prerequisite for more individualized home care visits.

The communication in home care showed a higher positive affect tone and was primarily socio emotional. These results may be expected because home care contains everyday talk and social interaction and is similar to previous research on cancer patients in home care [46]. The social and interpersonal contacts are described as important and valued by older persons receiving home care [4]. The global affect ratings further revealed that nurses often showed responsiveness, interactivity and interest. They were rarely hurried or dominant towards the older persons. The ability of nurses to listen and to be attentive to the narratives of others are important [16, 30, 31] and connected to the alleviation of suffering [31, 32].

No significant associations were found between the degree of patient-centeredness and the personal characteristics of the participants, such as gender or age. The lack of gender effects differs from previous research on gender effects and patient-centeredness in patient-physician consultations in primary- or hospital care [37, 38]. This may be due to the different care contexts. Our sample also included a smaller number of male participants. A more equal division of males and females might have rendered a different result. However, females are in majority among healthcare providers, in Sweden as in many other countries.

Another possible limitation is related to age; all RN participants were over 39 years of age. Hence, possible age-related differences between younger or older RNs are lacking in the data. The variables used in this study did not fully explain the degree of patient-centeredness in the different GLMM models. There are additional variables of importance for patient-centeredness in home care communication not covered in this study, such as touch, gaze or body position. Gorawarra Bhat et al. have explored the consistency between verbal and non-verbal communication between older persons and physicians in a Norwegian setting. They found that physicians frequently showed non-consistent behavior in challenging situations. This may be perceived as either alleviating or increasing distress, depending on the patient’s needs [47].

Future research on patient-centered communication in home care might benefit from including a more equal representation of age and gender, as well as variables, such as working life experience, how long the RN has cared for the older person, social status or the care needs of the older person. There are further limitations regarding the generalizability of the results as the total number of audio recordings and participants are limited, and the audio recordings are from a specific Swedish region and care context. Another limitation of generalizability is the recruitment of a convenience sample. Convenience sample may cause selection bias and a sample that is not representative of the larger population. However, the present study was an explorative study and not intended for theory testing, and the results can help others to further explore the degree of patient-centeredness in home care communication, as well as patient-nurse interactions in other care contexts. These data further show how communication contains both task-oriented, as well as socio-emotional utterances, which are important for all health care communication and for viewing the unique person and his/her individual needs.

### Conclusion

The degree of patient-centeredness was high, and the global affect was foremost positive. Longer home care visits revealed a higher degree of patient-centered communication, with a peak in visits that were 8–9 min long. Otherwise, the degree varied, and some longer visits contained a similar degree of patient-centeredness as in shorter visits. These findings can be used when organizing home care services, and for education and training purposes.

## Data Availability

The datasets generated and/or analyzed during the current study are not publicly available due to ethical reasons and the right to confidentiality for recorded persons but are available from the corresponding author on reasonable request.

## Abbreviations

GLMM: Generalized Linear Mixed Model
RIAS: Roter Interactional Analysis System
RN: Registered nurse
